# The Acute Effects of a Relative Dose of Pre-Sleep Protein on Recovery Following Evening Resistance Exercise in Active Young Men

**DOI:** 10.3390/sports9040044

**Published:** 2021-03-26

**Authors:** Juliana V. Costa, J. Max Michel, Takudzwa A. Madzima

**Affiliations:** 1 Energy Metabolism and Body Composition Laboratory, Department of Exercise Science, Elon University, 100 Campus Drive, Elon, NC 27244, USA; jcosta@elon.edu; 2 Department of Health and Exercise Science, Wake Forest University, 1834 Wake Forest Road, Winston-Salem, NC 27101, USA; michjm20@wfu.edu

**Keywords:** pre-sleep protein, recovery, appetite, resistance exercise, supplement

## Abstract

The purpose of the present study was to assess the acute effects of pre-sleep consumption of isocaloric casein protein (CP), CP and whey protein (BLEND), or non-caloric control (CTRL) at a dose relative to lean body mass (LBM) on recovery following an evening lower-body resistance exercise (RE) bout. Fifteen active and previously resistance-trained males (age: 21 ± 1 years, body fat: 14.2 ± 2.7%) participated in this randomized, single-blind, crossover study. Participants performed an evening lower-body RE bout and were provided with 0.4 g/kg/LBM of whey protein (WP) supplement post-RE. A single dose of 0.6 g/kg/LBM of CP, 0.4 g/kg/LBM of CP and 0.2 g/kg/LBM WP (BLEND), or CTRL was consumed 30 min prior to sleep. Measurements of perceived recovery (visual analogue scales (VAS) for recovery, soreness, and fatigue), appetite (VAS for hunger, satiety, and desire to eat), as well as pressure-pain threshold (dolorimeter), average power, and peak torque (isokinetic dynamometry) of the right thigh muscles were assessed the following morning. Main effects of time were seen for all recovery variables (perceived recovery: F_2,28_ = 96.753, *p* < 0.001, h_p_^2^ = 0.874; perceived fatigue: F_2,28_ = 76.775, *p* < 0.001; h_p_^2^ = 0.846; perceived soreness: F_2,28_ = 111.967, *p* < 0.001; h_p_^2^ = 0.889). A main effect of supplement was only seen for perceived recovery (F_2,28_ = 4.869; *p* = 0.015; h_p_^2^ = 0.258), with recovery being 6.10 ± 2.58 mm greater in CP vs. BLEND (*p* = 0.033) and 7.51 ± 2.28 mm greater in CP than CTRL (*p* = 0.005). No main effects of supplement were seen in measures of perceived soreness, or fatigue (F_2,28_ ≤ 2.291; *p* > 0.120; h_p_^2^ ≤ 0.141). No differences between supplements were found in perceived next-morning hunger (*p* = 0.06), satiety (*p* ≥ 0.227), or desire to eat (*p* = 0.528). Main effects of supplement were seen between BLEND and CP vs. CTRL for measures of pain-pressure threshold at the rectus femoris (F_2,28_ = 9.377; *p* = 0.001; h_p_^2^ = 0.401), the vastus lateralis (F_2,28_ = 10.887; *p* < 0.001; h_p_^2^ = 0.437), and the vastus medialis (F_2,28_ = 12.113, *p* < 0.001; h_p_^2^ = 0.464). Values of peak torque and average power were similar between all supplement groups at 60°/sec (F_1.309,18.327_ ≤ 1.994; *p* ≥ 0.173; h_p_^2^ ≤ 0.125), 180°/s (F_2,28_ ≤ 1.221; *p* ≥ 0.310; h_p_^2^ ≤ 0.080), and 300°/sec (F_2,28_ ≤ 2.854; *p* ≥ 0.074; h_p_^2^ ≤ 0.169). Pre-sleep consumption of CP and BLEND at a dose relative to LBM may enhance perceived overnight recovery to a greater extent than CTRL as a result of less muscle soreness the following morning after an acute evening RE bout.

## 1. Introduction

In the last few years, nutrient timing research has recognized the positive physiological benefits of pre-sleep consumption of protein within 30 min of sleep [1,2,3,4,5]. Among the benefits associated with pre-sleep feeding, recent studies suggest that this strategy can enhance overnight recovery of muscle function after evening exercise [3,6,7]. Protein feeding accelerates the remodeling process by inducing muscle protein synthesis (MPS) [8,9]. It has been established that active individuals should aim to increase the traditional recommended daily allowance of 0.8 g/kg/day [10] to about 1.4–2.0 g protein/kg/day for positive muscle protein balance [11]. Although this daily amount of protein could be attained through the consumption of whole foods (i.e., chicken, fish, black beans, etc.), it may not be convenient due to meal preparation time, timing of exercise, or athletic practices. For this reason, protein-rich supplements consumed in addition to whole foods are a common strategy used by several populations, including active individuals, to reach the daily recommended values of protein intake [6].

In addition to the quantity of protein consumed, quality is also an important factor to consider prior to acquiring a supplement. Previous research has recognized how a protein’s structure affects its speed of absorption [12], which impacts whole-body protein synthesis and breakdown [13]. Among the most widely studied protein supplements are the milk proteins casein (CP) and whey (WP) [1,2,6,7,9]. WP is a fast-acting protein, while CP is a slow-acting protein [10]. After ingestion of WP, there is a rapid but short-term rise in blood amino acids, which are the building blocks for muscle proteins [10,14]. CP, on the other hand, clots in the stomach, delaying the release of amino acids and inducing a prolonged, but moderate rise of amino acids within the blood [13].

The majority of previous pre-sleep studies primarily compared the effects of CP to a non-caloric placebo or carbohydrates on overnight recovery after exercise [6,7,15]. Overall, these studies utilizing pre-sleep CP at doses of 40–50 g reported enhanced overnight recovery from an evening exercise bout [6,7,15]. However, to date, no studies have investigated the effects on overnight recovery of a CP and WP blend (BLEND) compared to CP alone when consumed prior to sleep. It is plausible that BLEND could be more advantageous than consuming CP alone for the following reasons. First, WP has a higher proportion of essential amino acids (EAAs), which are critical for stimulating MPS and cannot be synthesized in the body [11,16] and thus must be obtained through food sources. Second, WP contains a greater amount of the amino acid leucine, which is a branched chain amino acid thought to be the primary initiator of MPS [10,16,17]. Lastly, the fast digestion nature of WP may be optimal for providing a rapid increase in blood amino acids during the first few hours of sleep, and the additional CP may sustain the circulating amino acid levels during a prolonged overnight period [17].

Although BLEND has not been tested in pre-sleep studies, a daytime study showed that a recovery drink composed of 80% CP and 20% WP provided both fast and slow delivery of amino acids to the muscle, attenuating losses in muscle function and improving recovery to a greater extent when compared to a carbohydrate drink [17]. Additionally, there is little to no research that uses a relative dose of pre-sleep protein. However, it has been suggested that pre-sleep protein relative to lean body mass may be needed to accommodate people’s varying weights and body compositions [2]. For this reason, this study compares a relative dose of (1) 0.6 g/kg/lean body mass (LBM) of CP, (2) 0.4 g/kg/LBM CP + 0.2 g/kg/LBM WP, and (3) a non-caloric control (CTRL) to deduce the most effective way of increasing overnight muscle recovery.

Therefore, the purpose of the present study is to assess effects of consuming CP and BLEND at a dose relative to LBM on appetite, recovery, soreness, and performance. Based on studies showing similar results, we hypothesize that there is no difference between supplement groups in measures of perceived appetite. Conversely, we hypothesize that there will be differences between supplement groups in measures of muscular performance, with CP and BLEND outperforming CTRL. We additionally hypothesize that CP and BLEND are superior to CTRL in measures of self-assessed recovery (perceived fatigue, soreness, and recovery) and assessments of pain-pressure threshold.

## 2. Materials and Methods

### 2.1. Participants

Fifteen physically active and previously resistance-trained college-aged males (age: 20.8 ± 0.7 years; height: 180.5 ± 5.4 cm; weight: 78.1 ± 6.6 kg; body fat %: 14 ± 2.7%; lean body mass: 62.7 ± 4.1 kg; BMI: 23.7 ± 1.9 kg m^−2^) participated in this study. Participants were recruited via flyers distributed throughout the Elon Koury Center (Elon, NC, USA), as well as via social media interaction and direct contact with participants once individuals were established as interested in the study. Participants were considered physically active if they engaged in vigorous physical activity (including both aerobic and resistance training) 3+ days per week for at least 30 min for the prior three months. Physical activity was determined via self-report. Participants were excluded if they had uncontrolled hypertension (BP > 160/100 mmHg), were taking blood pressure medication, or had been diagnosed with cardiovascular disease, diabetes, thyroid, or kidney disease. Participants who met inclusion criteria were invited to participate in the study and provided informed consent, completed the PARQ+ physical activity questionnaire, and a medical history questionnaire.

Participants were asked to refrain from taking any nutritional supplements (excluding multivitamins), alcohol, and caffeine for the duration of the study. Additionally, they were asked to refrain from strenuous exercise not included in the study protocol 48 h prior to initial testing, and for the duration of the study. Participants were also instructed to maintain records of dietary intake beginning 72 h prior to their first evening exercise session and ceasing after the last testing session using the mobile application MyFitnessPal (MyFitnessPal, Inc.; Baltimore, MD, USA). This mobile application has been validated against paper-based food logs [18]. Participants were asked to use these dietary intake records to replicate their nutrient intake for each subsequent testing visit. Prior to the first supplement intake, participants were provided a log sheet in which they recorded notable times, including when the supplement was consumed, when they laid down for bed, when they fell asleep, and when they woke up the following morning. The present study was conducted according to the guidelines laid down in the Declaration of Helsinki, and all procedures involving human participants were approved by the Elon University Institutional Review Board (Protocol #20-002, 3 June 2019.). Written informed consent was obtained before participation in the study.

### 2.2. Study Design

**Figure 1:**
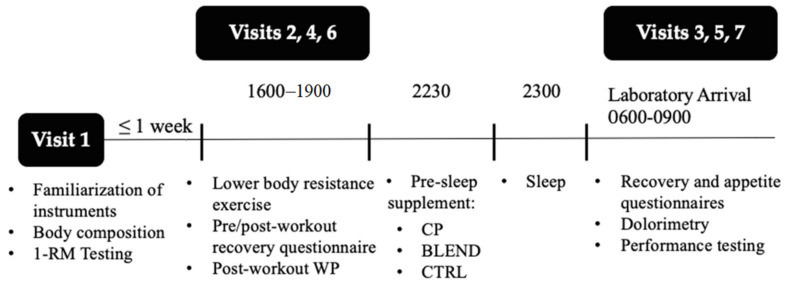
Experimental design.

The present study followed a randomized, single-blind, crossover design (Figure 1). During the first laboratory visit (familiarization), participants’ body composition was assessed via non-invasive Biodynamics™ bioelectric impedance analysis (Biodynamics Corp., Shoreline, WA, USA). Participants were familiarized with exercises included in the resistance exercise protocol, and 1 repetition maximums (1-RM) were assessed for both the leg extension and the horizontal leg press. Baseline measures of peak torque and average power were assessed via Biodex™ isokinetic dynamometry (Biodex System 4, Biodex Medical Systems, Inc., Shirley, NY, USA) in order to familiarize participants with the system. After the first laboratory visit, participants completed 3 experimental trials separated by at least 72 h. A trial is denoted by 2 subsequent laboratory visits. Visits 2, 4, and 6 consisted of the completion of resistance exercise protocol and visual analogue scale (VAS) measurements of recovery. Visits 3, 5, and 7 consisted of VAS measurements of recovery and appetite, isokinetic dynamometry, and pain-pressure threshold. The average time between experimental trials was 111 h ± 40. All visits are described in greater detail below.

### 2.3. Protein Supplements

At cessation of each evening exercise session, participants were given a post-workout supplement of whey protein standardized to 0.4 g/kg/lean body mass (LBM) to be consumed on site. This supplement was consumed in order to enhance ecological validity as well as provide standardized post-exercise nutrient intake to isolate the effects of prescribed pre-sleep supplement. Then, participants were randomly assigned via computer algorithm 1 of 3 supplements to consume prior to sleep: (1) 0.6 g/kg/LBM of CP (CP), (2) 0.4 g/kg/LBM CP + 0.2 g/kg/LBM WP (BLEND), and (3) a non-caloric control (CTRL) (Propel Zero™, The Gatorade Company, Chicago, IL, USA). CP and WP were sourced from Optimum Nutrition^®^ (Aurora, IL, USA). One scoop of CP (36.5 g; 100% Gold Standard) comprised 130 calories, 0.5 g total fat, 7 g carbohydrates, and 24 g proteins, whereas one scoop of WP (31 g; 100% Gold Standard) comprised 110 calories, 1 g total fat, 2 g carbohydrates, and 24 g protein. Both CP and WP were vanilla flavored and had identical texture to ensure that participants were blinded to each experimental trial. Powdered CP, BLEND, and CTRL were labelled A, B, and C and packaged by an external investigator who was not involved in data collection. Although the non-caloric CTRL was in powdered form, its color, flavor, and consistency differed from those of the CP and BLEND. Participants were given each supplement in a blender bottle and were instructed to consume it at home with 12 oz of water at least 1 h following consumption of their last meal and 30 min prior to sleep. The following morning, participants returned to the lab with their blender bottle and a log with the approximate times of supplement consumption and sleep.

### 2.4. Experimental Trials (Visits 2–7)

#### 2.4.1. Evening Visits (Visits 2, 4, 6)

Participants arrived at the lab between 3:30 p.m. and 7 p.m. Subjective assessments of recovery (recovery, soreness, and fatigue) were collected using a validated visual analogue scale (VAS) [19]. The VAS is a 100-mm horizontal scale that allows participants to recognize their subjective feeling of recovery between opposite ends of a scale, ranging from “not at all” to “extremely” anchored at 0 mm and 100 mm, respectively. Participants were instructed to draw a single line perpendicular to the 100 mm line at their perceived level of feeling. This distance was then measured using a standard ruler and recorded for analysis, with higher scores indicating a greater sensation. Importantly, initial VAS measures of recovery are referred to as T_1_. After subjective assessments of recovery were collected, participants were escorted to the Elon University Koury Athletic Center by the research investigator who would guide them through a lower-body resistance exercise. Participants completed a warm-up in which they pedaled a cycle ergometer for 5 min at a rate of perceived exertion of 9 on a scale of 6–20 [20]. After warm-up was completed, participants performed 8 sets of 8 repetitions on the horizontal leg press machine followed by identical set x repetition structure for the leg extension. The 8 sets for both exercises were partitioned as such: one set at 55%, one at 65%, and six at 75% of their 1 repetition maximum. There was a 2 min rest period between sets and a 5 min rest period between exercises. This resistance exercise protocol was chosen based on a similar pre-sleep nutrient study’s protocol employed by Res et al. [7]. Parameters for a successful repetition of the leg press were that the participant must reach 90° of flexion at the knee during the eccentric portion of the lift and reach extension without locking the knee during the concentric portion. Parameters for successful completion of the leg extension were that the participant must fully extend the knee to lockout and return to at least 90 degrees of flexion during the eccentric phase of the lift. Participants performed each repetition following a metronome cadence of 30 beats per minute to ensure standardization between sets and participants. All training sessions were monitored by the same investigator. After the completion of the exercise bout, participants completed an additional subjective assessment of recovery (VAS, T_2_) and were given the standardized post-workout supplement, pre-sleep supplement, and the sleep times log sheet.

#### 2.4.2. Morning Assessments (Visits 3, 5, 7)

Participants returned to the lab fasted the morning after each evening exercise bout between 07:00 and 09:00 a.m. The sleep log was collected, and participants were asked to complete a VAS for both recovery (T_3_) and appetite. Appetite was assessed through participants’ perceived hunger, satiety, and desire to eat. VAS protocol and measurement was the same as described previously. Additionally, participants completed an assessment of muscular function by performing knee flexion and extension on a Biodex™ isokinetic dynamometer. Participants were seated and strapped at the trunk and pelvis into the Biodex™ isokinetic dynamometer (Biodex System 4, Biodex Medical Systems, Inc., Shirley, NY, USA), ensuring that their hip angle was approximately 90°. The input axis of the dynamometer was aligned with the lateral epicondyle of the femur of the participants preferred leg. Participants completed isokinetic testing at 60°/s, 180°/s, and 300°/s, wherein measures of peak torque and average power of both the flexion and extension phase were recorded. Multiple velocities were tested on the basis that participants could have been differentially recovered for varying activities. According to the force-velocity relationship, different velocities of contraction represent differing functional outcomes; therefore, it is reasonable that a range of velocities be interrogated to assess potentially differential recovery. Participants were subjected to a handheld dolorimeter in which assessments of pain-pressure threshold were collected. Pain-pressure threshold was determined by pressing the dolorimeter against the vastus medialis, rectus femoris, and vastus lateralis sequentially, with participants instructed to indicate vocally when the stimulus was deemed painful. This process was repeated until all three leg muscles had been tested. The dolorimeter provided a reading of force output in Newtons that was recorded and stored for analysis. Importantly, testing occurred at ~12 h post-evening exercise bout in order to evaluate short term recovery differences as a result of differential supplementation. All morning testing was performed and monitored by a trained investigator (JVC).

### 2.5. Statistical Analysis

Following the completion of the study, data analyses were conducted using IBM SPSS, Version 26.0 (Chicago, IL, USA). Normality of all dependent variables was formally assessed using a Shapiro-Wilk test. If dependent variables violated assumptions of normality, log10 and square-root transformations were attempted. If transformations resolved violations of normality, parametric tests were used to analyze data; however, if data were unable to be transformed, a non-parametric equivalent test was used. Data were also analyzed for sphericity using Mauchly’s test of sphericity when using a within-factors test. Subjective assessments of recovery (recovery, fatigue, and soreness) were assessed via within-factors two-way supplement × time ANOVA wherein data were assessed for main effects of time and supplement, and significant interactions between supplement and time. Estimated marginal means were generated after determination of no significant interaction and used to perform pairwise comparisons across group and time. Subjective assessments of appetite (hunger, satiety, and desire to eat), isokinetic dynamometry, and pressure–pain threshold data were analyzed via within-factors ANOVA. Estimated marginal means were once again generated and used to perform pairwise comparisons across groups. Statistical significance was set a priori at *p* = 0.05.

## 3. Results

### 3.1. Training Volume

There were no statistically significant differences in total volume load performed by participants across supplement groups (*p* > 0.05). Across all training sessions, CTRL averaged 10,973 ± 619.59 kg, BLEND averaged 10,799 ± 717.91 kg, and CP averaged 11,049.20 ± 645.22 kg.

### 3.2. Nutrient Intake

There were no statistically significant differences in macronutrient intake or overall energy intake between supplement trials. Analysis of dietary logs revealed that participants in the CTRL condition consumed an average of 1887 ± 409 kcals (107 ± 41 g of protein, 202 ± 45 g of carbohydrates, 72 ± 30 g of fat), participants in the BLEND condition consumed an average of 1802 ± 453 kcals (105 ± 34 g of protein, 193 ± 70 g of carbohydrates, 68 ± 22 g of fat), and participants in the CP condition consumed an average of 1696 ± 404 kcal (108 ± 35 g of protein, 193 ± 70 g of carbohydrates, 68 ± 34 g of fat). Importantly, macronutrient and energy intake data do not include the prescribed supplement or post-exercise supplement.

### 3.3. Assessment of Recovery

**Figure 2:**
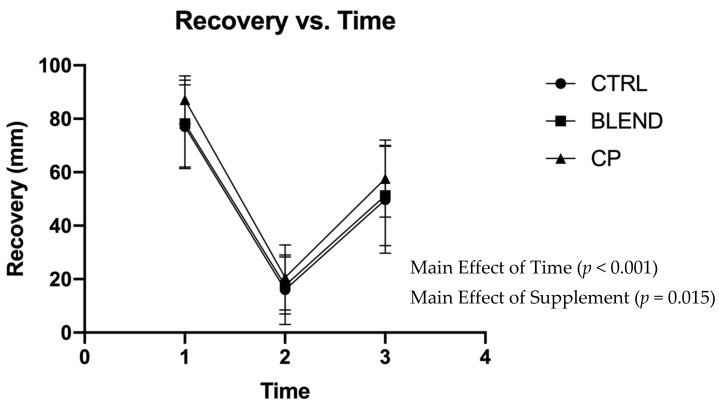
Visual analogue scale of recovery across time points. CTRL: control; BLEND: casein protein and whey protein; CP: casein protein. Data are presented as means with standard deviation as error bars. [T_1_: pre-resistance exercise; T_2_: post-resistance exercise; T_3_: morning-after].

**Figure 3:**
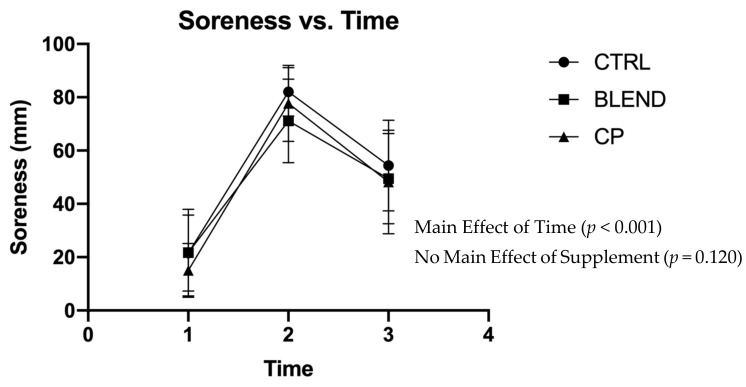
Visual analogue scale of soreness across time points. CTRL: control; BLEND: casein protein and whey protein; CP: casein protein. Data are presented as means with standard deviation as error bars. [T_1_: pre-resistance exercise; T_2_: post-resistance exercise; T_3_: morning-after].

**Figure 4:**
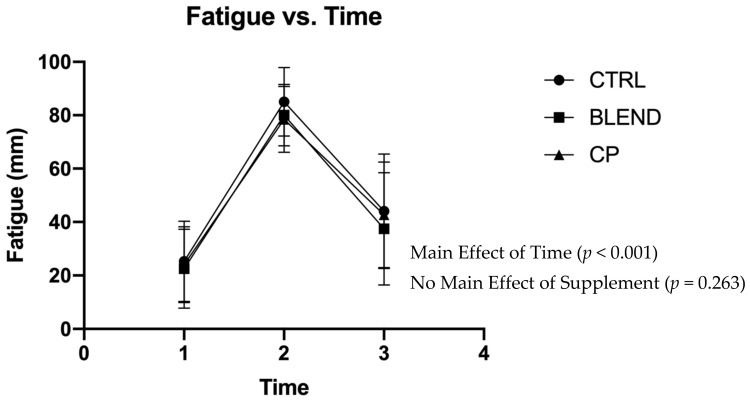
Visual analogue scale of fatigue across time points. CTRL: control; BLEND: casein protein and whey protein; CP: casein protein. Data are presented as means with standard deviation as error bars. [T_1_: pre-resistance exercise; T_2_: post-resistance exercise; T_3_: morning-after].

Subjective assessments of recovery, soreness, and fatigue were analyzed via repeated measures two-way ANOVA. Data are presented in Figure 2, Figure 3 and Figure 4. There were no significant supplement x time interactions among recovery (F_4,56_ = 0.383; *p* = 0.820; partial eta squared (η_p_^2^) = 0.027), soreness (F_4,56_ = 1.063; *p* = 0.383; η_p_^2^ = 0.071), or fatigue (F_4,56_ = 0.358; *p* = 0.837; η_p_^2^ = 0.025). Analysis revealed no main effect of supplement on self-assessed fatigue (F_2,28_ = 1.399; *p* = 0.263; η_p_^2^ = 0.091) or soreness (F_2,28_ = 2.291; *p* = 0.120; η_p_^2^ = 0.141). Pairwise comparisons revealed no statistically significant differences between supplement groups for self-assessed fatigue (*p* >0.05) and soreness (*p* > 0.05). There was, however, a main effect of supplement on self-assessed recovery (F_2,28_ = 4.869; *p* = 0.015; η_p_^2^ = 0.258). Pairwise comparisons revealed a significant difference between CP vs. CTRL (*p* = 0.005) and CP vs. BLEND (*p* = 0.033), with recovery being 6.10 ± 2.58 mm greater for CP than the blend, and 7.51 ± 2.28 mm greater for CP than CTRL. As expected, there was a main effect of time for recovery (F_2,28_ = 96.753; *p* < 0.001; η_p_^2^ = 0.874), fatigue (F_2,28_ = 76.775, *p* < 0.001; η_p_^2^ = 0.846), and soreness (F_2,28_ = 111.967, *p* < 0.001; η_p_^2^ = 0.889).

### 3.4. Assessment of Appetite

Subjective assessments of hunger, satiety, and desire to eat were analyzed via repeated measures ANOVA, or Friedman’s Test if data were unable to be transformed to normality. Data are presented in Table 1. There were no statistically significant differences between hunger (*p* = 0.06), satiety (*p* ≥ 0.227), or desire to eat (*p* = 0.528) among the CP, BLEND, and CTRL groups. However, although not statistically significant, the CP group reported more hunger (52.20 ± 4.45 mm) than the BLEND group (39.93 ± 4.12 mm) and the CTRL group (46.53 ± 4.70 mm).

### 3.5. Pressure-Pain Threshold

**Figure 5:**
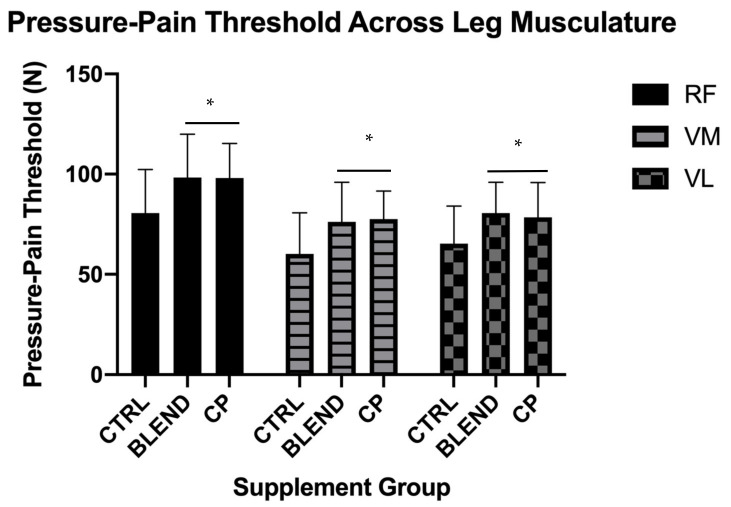
Pressure-pain threshold assessed across leg musculature at the morning-after time point. CTRL: control; BLEND: casein protein and whey protein; CP: casein protein; RF: rectus femoris; VM: vastus medialis; VL: vastus lateralis; * denotes a significant difference between either BLEND or CP and CTRL. Data are presented as means with standard deviation as error bars.

Data collected via dolorimeter to assess pressure-pain threshold were analyzed via within-factors ANOVA. Data are presented in Figure 5. There was a main effect of supplement at the rectus femoris (RF) (F_2,28_ = 9.377, *p* = 0.001; η_p_^2^ = 0.401), the vastus lateralis (VL) (F_2,28_ = 10.887, *p* < 0.001; η_p_^2^ = 0.437), and the vastus medialis (VM) (F_2,28_ = 12.113, *p* < 0.001; η_p_^2^ = 0.464). Pairwise comparisons revealed a significant difference between CP vs. CTRL at the RF (*p* = 0.005), the VL (*p* = 0.007), and the VM (*p* = 0.003). These comparisons also revealed a significant difference between BLEND vs. CTRL at the RF (*p* = 0.002), the VL (*p* < 0.001), and the VM (*p* = 0.002). There was, however, no significant difference between CP vs. BLEND at the RF (*p* = 0.952), the VL (*p* = 0.523), or the VM (*p* = 0.471).

### 3.6. Isokinetic Dynamometry

Isokinetic dynamometry data were assessed via within-factors ANOVA or Friedman’s Test if data were unable to be transformed to normality. Data are presented in Table 2. No main effects of supplement were seen for measures of peak torque and average power at 60°/s (F_1.309,18.327_ ≤ 1.994; *p* ≥ 0.173; η_p_^2^ ≤ 0.125), 180°/s (F_2,28_ ≤ 1.221; *p* ≥ 0.310; η_p_^2^ ≤ 0.080), or 300°/s (F_2,28_ ≤ 2.854; *p* ≥ 0.074; η_p_^2^ ≤ 0.169).

## 4. Discussion

The present study is the first to examine the effects of consuming a CP or BLEND supplement at a dose relative to LBM pre-sleep on perceived recovery, next-morning perceived appetite, soreness, and performance following an evening lower-body resistance exercise bout. Contrary to our hypothesis, there were no significant differences in measures of perceived soreness or perceived fatigue across supplement groups. There was, however, a main effect of supplement on perceived recovery with CP outperforming BLEND and CTRL. This is somewhat consistent with hypotheses, as we hypothesized that CP and BLEND would outperform CTRL. Consistent with our hypotheses, no difference was seen across supplement groups for any measure of perceived appetite. Additionally, consistent with our hypothesis, consumption of CP or BLEND resulted in decreased soreness, as assessed by measurement of pain-pressure threshold, when compared to CTRL in physically active males. Finally, contrary to hypotheses, there were largely no differences across supplement groups in measures of muscle performance as assessed via isokinetic dynamometry in physically active males.

### 4.1. Assessment of Recovery

Analysis of perceived recovery revealed a main effect of supplement, with CP outperforming BLEND and CTRL. Therefore, findings of a main effect of supplement on perceived recovery are consistent with previous findings of enhanced overall recovery following a bout of athletic performance (soccer game) at nighttime in young male athletes [6]. Similarly, enhanced recovery was seen in a study [7] whose findings suggest that pre-sleep protein consumption in conjunction with bouts of resistance exercise increase mixed-muscle protein fractional synthetic rate (FSR) when compared to a placebo in young active men. Conversely, our finding of a main effect of supplement on perceived recovery is contrary to findings [21] that suggest pre-sleep ingestion of protein does not functionally improve recovery in young active males when exercise is performed in the morning. Importantly however, this study by Apweiler et al. assessed recovery functionally and not perceptually. Additionally, participants performed a muscle damaging protocol, consisting primarily of eccentric contractions, in the morning hours and consumed either CP or CTRL pre-sleep the same day. Our finding of no difference across groups in measures of perceived soreness is supported by previous work [22] that found no differences existed between pre-sleep whey protein consumption, plant protein consumption, or placebo in measures of perceived soreness the morning following a bout of muscle damaging exercise. Similarly, the lack of difference among supplement groups in measures of perceived fatigue is supported by lack of differences seen in related measures as previously mentioned and reported [21,22]. It is possible that pre-sleep protein plays a role in overnight recovery, both functionally and perceptually, potentially due to its role in enhancing circulating amino acid levels [7]. Additionally, it has been reported that resistance exercise in conjunction with whey protein induces a greater anabolic response than whey protein alone [15]; however, the interplay that exists between pre-sleep protein type, resistance exercise timing, and overall MPS remains to be determined, specifically as it relates to measures of recovery in young men.

### 4.2. Assessment of Appetite

Our findings suggest no difference exists between pre-sleep CP, BLEND, and CTRL in subjective measures of next-morning appetite and are consistent with findings of similar studies [1,2,23]. In contrast to these findings, a previous study [5] found that pre-sleep consumption of either carbohydrate supplement, whey protein supplement, or casein protein supplement decreases next-morning desire to eat while increasing next-morning satiety. This difference in findings could be attributable to the population being examined, as in the aforementioned study, the population was overweight or obese sedentary women. Additionally, the same group reported an increased desire to eat in overweight or obese men consuming CP pre-sleep as opposed to a non-caloric control [24]. No differences in satiety or hunger were seen. Given the aforementioned findings, the effect of population, exercise timing, and pre-sleep supplement type on next-morning measures of subjective appetite remains unclear. It is possible that these factors play a role in measures of subjective appetite assessment; however, it has been shown repeatedly that next-morning subjective appetite assessment does not differ between supplement groups in a young, active population.

### 4.3. Pain-Pressure Threshold

Consistent with our hypothesis, pain-pressure threshold was significantly higher at the VL, VM, and RF in CP and BLEND as compared to CTRL. Utilization of measurements of pain-pressure threshold in a comparable design is somewhat limited; however, one similar study [21] reported only main effects of time, where pain-pressure threshold decreased over the course of 48 h regardless of supplement group. Given that in the present study, differences were seen between both BLEND vs. CTRL and CP vs. CTRL, it is possible that this measure was affected by lower rates of whole-body overnight protein breakdown, enhanced overnight whole-body protein synthesis, and enhanced overnight muscle FSR [7,25]. Additionally, the interaction between resistance exercise and pre-sleep protein consumption has been shown to enhance myofibrillar protein synthesis [15]. These enhanced rates of protein accretion due to both pre-sleep protein consumption and resistance exercise could impact this measure of soreness, and further investigation into this relationship is warranted.

### 4.4. Isokinetic Dynamometry

No main effects of supplement were seen in measures of isokinetic dynamometry. This finding is in agreement with previous findings that saw no main effects of supplement group in measures of peak torque after consumption of pre-sleep protein and a muscle damaging protocol [22].

### 4.5. Experimental Considerations

This study is limited in the fact that mechanistic targets of recovery due to pre-sleep protein were not collected, thus limiting ability to infer causation. Similarly, it was the original intent of the study to collect and analyze salivary samples to provide measures of testosterone and cortisol; however, due to disruption regarding the COVID-19 pandemic, these samples were unable to be analyzed. The addition of a standardized post-workout supplement constitutes another limitation of this study. It is possible, given that training occurred in the evening hours as late as 1900 h, that this supplementation and the possible subsequent meal that participants consumed influenced the effect of the prescribed pre-sleep supplement. It is additionally possible that findings underestimate the true impact of the aforementioned prescribed pre-sleep supplement. Another limitation of the current study is the sample that was studied, as findings are likely not generalizable to a sedentary, elderly, or female population given that participants were young, active males. Another limitation of the present study is reliance on self-reporting of nutrient intake. While MyFitnessPal™ is a validated measure, there is inherently variability in self-report measures that could present low reliability in some cases. There is also potential for a varied training background among participants included in the study that could account for variance of findings. Finally, it is possible that outcomes were affected by order of supplementation among trials. Given that there were 15 participants, analysis of effects of order remains underpowered, and this remains an unresolved limitation of this study.

### 4.6. Additional Considerations

Despite certain limitations, the present study does present a crossover design that provided a basis for a within-individual analysis of main outcome variables. The crossover design employed is not, however, without its limitations. These limitations are chiefly the possibility to introduce effects of order (discussed above), and the potential for carry-over effects to confound results. As mentioned previously, this study was not designed to be sufficiently powered to detect an effect of order; there was, however, a washout period of at least 72 h in which subjects were not prescribed a supplement in the hopes of eliminating any potential carry-over effects. This study is additionally the first of its kind to analyze pre-sleep protein consumption at a dose relative to LBM after a bout of resistance exercise and provides analysis of both objective and subjective measures of overall next-morning recovery. Further investigation into mechanistic targets (i.e., muscle protein synthesis, net protein balance, muscle protein breakdown) and their effect on these measures of subjective and objective recovery is warranted at a dose of pre-sleep protein relative to LBM.

## 5. Conclusions

Contrary to our hypothesis, next-morning perceived soreness and fatigue were not enhanced by consuming CP or BLEND as opposed to CTRL. However, next-morning perceived recovery was greater in CP compared to CTRL or BLEND. Additionally, as hypothesized, CP and BLEND led to less overall muscle soreness the morning after a bout of damaging muscle resistance exercise. Therefore, pre-sleep consumption of CP and BLEND at a dose relative to LBM may enhance perceptions of overnight recovery to a greater extent than CTRL as a result of less muscle soreness the morning after a bout of resistance exercise.

## Figures and Tables

**Table 1:** Visual analogue scale of hunger, satiety, and desire to eat (*n* = 15) (mean values with their standard deviations).

Appetite Variable	CTRL		BLEND		CP	
	Mean	SD	Mean	SD	Mean	SD
Hunger (mm)	46.53	18.21	39.93	15.95	52.20	17.21
Satiety (mm)	39.07	17.32	44.80	19.47	37.67	13.39
Desire to Eat (mm)	47.40	21.78	45.80	16.71	51.67	17.65

CTRL: control; BLEND: casein protein with whey protein; CP: casein protein.

**Table 2:** Isokinetic dynamometry data.

			CTRL		BLEND		CP	
			Mean	SD	Mean	SD	Mean	SD
**60** **°** **/s**	Average Power (Watts)	Extension	102.03	24.67	106.48	31.88	103.19	24.72
		Flexion	64.13	14.39	67.56	18.54	63.90	12.92
	Peak Torque (foot × lbs)	Extension	116.04	24.16	123.50	35.88	116.93	24.23
		Flexion	72.31	13.65	73.64	18.27	72.68	11.46
**180** **°** **/s**	Average Power (Watts)	Extension	174.53	54.48	183.79	61.42	185.53	60.14
		Flexion	105.18	32.99	111.50	35.21	111.37	31.59
	Peak Torque (foot × lbs)	Extension	80.90	21.80	81.82	22.42	81.83	20.71
		Flexion	49.86	10.20	54.23	18.36	51.73	10.80
**300** **°** **/s**	Average Power (Watts)	Extension	155.40	71.80	165.73	67.17	169.63	69.12
		Flexion	79.71	46.68	85.15	43.93	94.27	45.09
	Peak Torque (foot × lbs)	Extension	57.61	17.08	62.280	20.16	58.37	15.83
		Flexion	35.62	10.75	37.24	9.70	37.82	8.33

CTRL: control; BLEND: casein protein and whey protein; CP: casein protein. Extension: knee extension; Flexion: knee flexion. Data are presented as means and standard deviation.

## Data Availability

Data presented in this study are available on request from the corresponding author. The data are not publicly available due to privacy concerns regarding participant confidentiality.
